# Energy efficient group priority MAC protocol using hybrid Q-learning honey Badger Algorithm (QL-HBA) for IoT Networks

**DOI:** 10.1038/s41598-024-83234-5

**Published:** 2024-12-28

**Authors:** Ilayaraja Venkatachalam, Sirajudeen Ameer john, Jagadeesan Srinivasan, Senthilnathan Palaniappan, S. K. Somasundaram

**Affiliations:** 1 Department of IOT, School of Computer Science and Engineering, Vellore Institute of Technology, Vellore, India; 2 School of Computing Science and Engineering, VIT Bhopal University, Bhopal-Indore Highway, Kothrikalan, Sehore, 466114 Madhya Pradesh India; 3 Department of Software and Systems Engineering, School of Computer Science Engineering and Information Systems, Vellore Institute of Technology, Vellore, Tamil Nadu India; 4 Department of Analytics, School of Computer Science and Engineering, Vellore Institute of Technology, Vellore, India; 5 Department of Information Security, School of Computer Science and Engineering, Vellore Institute of Technology, Vellore, 632014 Tamil Nadu India

**Keywords:** Internet of things (IoT), Honey Badger Algorithm (HBA), Medium access control (MAC) protocol, Q-Learning, Group priority, Energy science and technology, Engineering, Mathematics and computing

## Abstract

In Internet of Things (IoT) networks, identifying the primary Medium Access Control (MAC) layer protocol which is suited for a service characteristic is necessary based on the requirements of the application. In this paper, we propose Energy Efficient and Group Priority MAC (EEGP-MAC) protocol using Hybrid Q-Learning Honey Badger Algorithm (QL-HBA) for IoT Networks. This algorithm employs reinforcement agents to select an environment based on predefined actions and tasks. It makes use of Q-learning method in Honey Badger Algorithm (HBA). In this algorithm, the PAN coordinator divides the network devices into multiple subgroups based on location, energy levels and the traffic type. In group priority assignment phase, a combined metric will be derived in terms of these parameters. Then a priority will be assigned to each group based on their combined metric. From each group, the optimal number of contention nodes will be selected using hybrid QL-HBA algorithm. The fitness function is derived in terms of the number of neighbours and total traffic loads of the nodes. Then transmission slots will be allotted to the group according to their group priority. The proposed EEGP-MAC protocol is implemented in NS3. Simulation results have shown that EEGP-MAC attains 11% lesser delay, 16% lesser energy consumption with 10% higher throughput, when compared to existing QL-DGMAC protocol, in various network sizes.

## Introduction

IoT gives billions of devices omnipresent wireless connectivity. IoT networks are often made up of massive clusters of devices that are dispersed spatially over large geographic areas. IoT enables the management and connection of many public services and infrastructure in smart cities^1,2^.

These IoT devices have a variety of sensors to measure, monitor, and report on some physical occurrences. Such IoT devices will produce massive volumes of data due to their dense geographical distribution. Whenever the transmission of data is inaccurate and causes incorrect actions, the IoT network’s accuracy suffers. By introducing redundant bits to the original information at the information level, fault tolerance and dependability can be improved^3^. For IoT applications supporting emergency applications, reliable data collection is a crucial concern. The BS receives the crucial data from the devices and processes it for decision-making.^4,5^.

IoT allows for a paradigm change from a manual method to an automated one. To maximize productivity, it organizes resources in a manner that is both cost and time efficient^6^. In IoT, numerous types of physical devices are being connected to each other and to a broader internet using enhanced communication technologies and electronics”. These physical items, for example, in agricultural, city, home automation, car and transportation, typically have the job of measuring and managing the smart environment with minimum human contact. Sensor nodes are often connected to each item in order to gather real-time data for analysis and decision-making. IoT has a lot of benefits, but it also comes with a lot of issues. IoT-enabled car processes that use wireless technology instead of wires to connect sensor nodes are becoming incredibly common.^7^.

The conventional Wireless Sensor Network (WSN) must be examined in order to enable effective communication for the IoT. Due to WSNs’ use of a proprietary MAC standard, transmission of data consumes less power. Current IoT gadgets, on the other hand, are well-suited to operating in higher-capability settings^8^.

Due to the nature of MAC protocols, fair and effective wireless resource sharing is impossible in the face of interference from other technologies. In the event that a battery dies, IoT devices will not be able to communicate with other network members^9^. Classifying and identifying the primary MAC layer protocols that are suited for a service characteristic is feasible based on the needs of the application^10^.

Contention-free protocols, contention-based protocols, and hybrid protocols all have a significant impact on the amount of energy consumed by the network. The TDMA technique is frequently used to regulate the channel in IoT networks to implement the contention-free protocol. Inefficient channel use or a limited network’s ability to scale are the results of using the TDMA technology, which is difficult to adjust for different network loads such as low or high. Transmission times are delayed when the beacon interval is increased because of this fixed length. CSMA/CA uses a contention-based mechanism to ensure that IoT devices can share the medium equally.

A high number of IoT devices or a problem with hidden nodes could lead to congestion in contention-based protocols. Congestion increases the number of retransmissions, which has an effect on the overall network performance in terms of channel usage and power consumption. Nodes under the CSMA protocol also use backoffs to avoid collisions (or interference) in high-density situations, which can lead to excessive delays. Small, intermittent data will be affected by these large delays.^12^.

To overcome the limitations of the aforesaid protocols and combine their benefits, a hybrid CSMA/CA-TDMA protocol might be effective in wireless networks, enhancing channel use and decreasing power consumption at the same moment.

### Research gaps and significant of the research

In^7^, the hybrid scheme combines the history and priority-based MAC procedures. History-based MAC utilizes the historical contention information to optimize the future contention window. Priority-based MAC assigns priority based on the time-criticality of the sensing data. However, energy efficiency is not ensured in this scheme.

In the protocol of^11^, the PAN coordinator divides the network devices into multiple subgroups based on the ID and location of the devices. But it did not consider the energy levels, type of data and traffic demands of the devices while grouping.

In QL-based DQMAC^13^, optimal number of contention nodes was determined by Q-learning algorithm. But it did not consider the total traffic loads of the nodes while determining the no. of contention nodes.

## Related works

### Standard MAC protocols of IoT

Many of the short-range protocols are based on the IEEE 802.15.4 standard’s PHY layer. There are several benefits to IEEE 802.11a over IEEE 802.15.4 in terms of throughput and energy utilization. The IEEE 802.15.4 PHY layer is used by the Wireless-HART protocol to improve its MAC layer with functionalities like frequency shifting and TDMA for medium access, increasing its capacity. Through a number of MAC layer techniques, IEEE 802.11ah considerably enhances the robustness and throughput of data transfer.

Because LoRa lacks power control and baud rate adjustment capabilities, its modulation is ineffective for long-range Internet of Things protocols that need high transmission rates and frequent broadcasts. In this regard, LoRaWAN, its successor, performs better than LoRa. LoRaWAN enhances the MAC layer by incorporating power control mechanisms, classifying operational modes of devices, and using adaptive data rates.

### MAC protocols found in the literature

MAC strategy developed by Arafatur Rahman and colleagues can be scaled up to meet various sensor-traffic quality of service requirements, as the researchers have shown^7^. The hybrid method tries to efficiently distribute network resources for smooth transmission from many sensors by combining history- and priority-based MAC. This method is based on MAC history. Packet collisions are reduced and average data delivery is accelerated when using a history-based MAC, which uses previous contention data to improve a near future contention window. For network planning, priority-based MAC prioritizes sensor data according to its time-criticality.

WLAN Aware Cognitive Medium Access Control Protocol for IoT Users (WAC-MAC) developed by Asfund Ausaf et al.^9^ uses energy detection based sensing, adaptive scheduling, and adaptive backoff to reduce interference with WSN and increase the network lifespan of IoT users. Packet reception rates can be improved while IoT node energy consumption is reduced using the WAC-MAC suggested herein.

Thair. A. Al-Janabi^11^ and his colleagues have devised a dynamic sleep/wake-up schedule that dynamically adjusts to changes in network load. The TDMA time slots are scheduled first, and then each slot is allocated to a set of competing devices using CSMA/CA. The base station (BS) broadcasts a scheduling table containing network grouping information to assist IoT devices in classifying themselves into wake-up and sleep groups. It allows for fewer collisions or failures in access to channels by letting only one group to compete for each slot. Three-dimensional Markov models are used to build stochastic behavior for the proposed adaptable sleep mode based on the hybrid MAC protocol.

Arjun Bakshi et al.^12^ characterize global interference statistics in terms of single-device operation in order to ensure low-delay high-reliability performance. They then create algorithms for allocating power rates. We tested EMIT’s performance against that of CSMA-based MAC protocols in order to confirm these theoretical hypotheses in the real world. EMIT has a significant advantage over CSMA in IoT traffic, according to their comparisons.

It was suggested by Chien-Min Wu et al.^13^ that an IoT-based distributed queueing MAC (DQMAC) protocol be developed. QL-based DQMAC proposes an ideal number of IoT nodes for contention. The Q-learning technique is used by each node to determine its own active rate. The active rate is used by each node to decide whether it will be active or in sleep mode during the next contention period. The likelihood of collision is reduced by finding the best IoT nodes in each contention phase. As a result of the smaller number of contentions, energy consumption and delay are reduced.

According to Abdellah Chehri et al.^14^, this technique might be used in e-health applications. This MAC protocol uses Dynamic Channel Coding (DCC) to mitigate interference in ultra-low radiated power ad hoc Ultra wide Band (UWB) networks. By enabling sources beyond the exclusion area to communicate, this protocol integrates the physical and MAC layers to improve throughput. DCC-MAC is updated in this study to include node energy usage and power level switching across nodes in real time.

Dynamic real-time applications and services need brand-new Quality of Service (QoS) architectures for the Point Coordination Function, according to Abbas Alnazir et al.^15^, ^16^ This work addresses the distributed coordination function (DCF) with improved distribution coordination function (EDCF) in stations and other situations to obtain the outcomes of throughput, retransmission attempts, latency, and data drop in stations and other scenarios. There are a few remote stations and one base station in the study’s specified distant LAN architecture. All distant stations are discovered to be capable of differentiating a broadcast from any other station using the provided intelligent framework.

We can scale throughput in an ultra-dense multi-cell random access network to its maximum level with only one AP and a sizable number of users according to Huifa Lin et al.^18^. Their protocol states that each user may independently transmit with a predetermined physical layer (PHY) data rate in an optimum manner if the intended signal power to the serving Access Point (AP) and the generating interference leakage power to other APs are both large enough. In a high Signal to Interference Ratio (SINR) regime, the optimum aggregate throughput scaling may be attained if the number of per-cell users is greater than a predetermined threshold.

## Proposed methodology

### Overview

In this paper, we propose Energy Efficient and Group Priority MAC protocol using Hybrid Q-Learning Honey Badger Algorithm (QL-HBA) for IoT Networks. In this algorithm, the PAN coordinator divides the network devices into multiple subgroups based on location, energy levels and the traffic type. In group priority assignment phase, a combined metric will be derived in terms of these parameters. Then a priority will be assigned to each group based on their combined metric. From each group, the optimal number of contention nodes will be selected using hybrid QL-HBA algorithm. The fitness function is derived in terms of the number of neighbours and total traffic loads of the nodes. Then transmission slots will be allotted to the group according to their group priority.

### System model

**Fig. 1:**
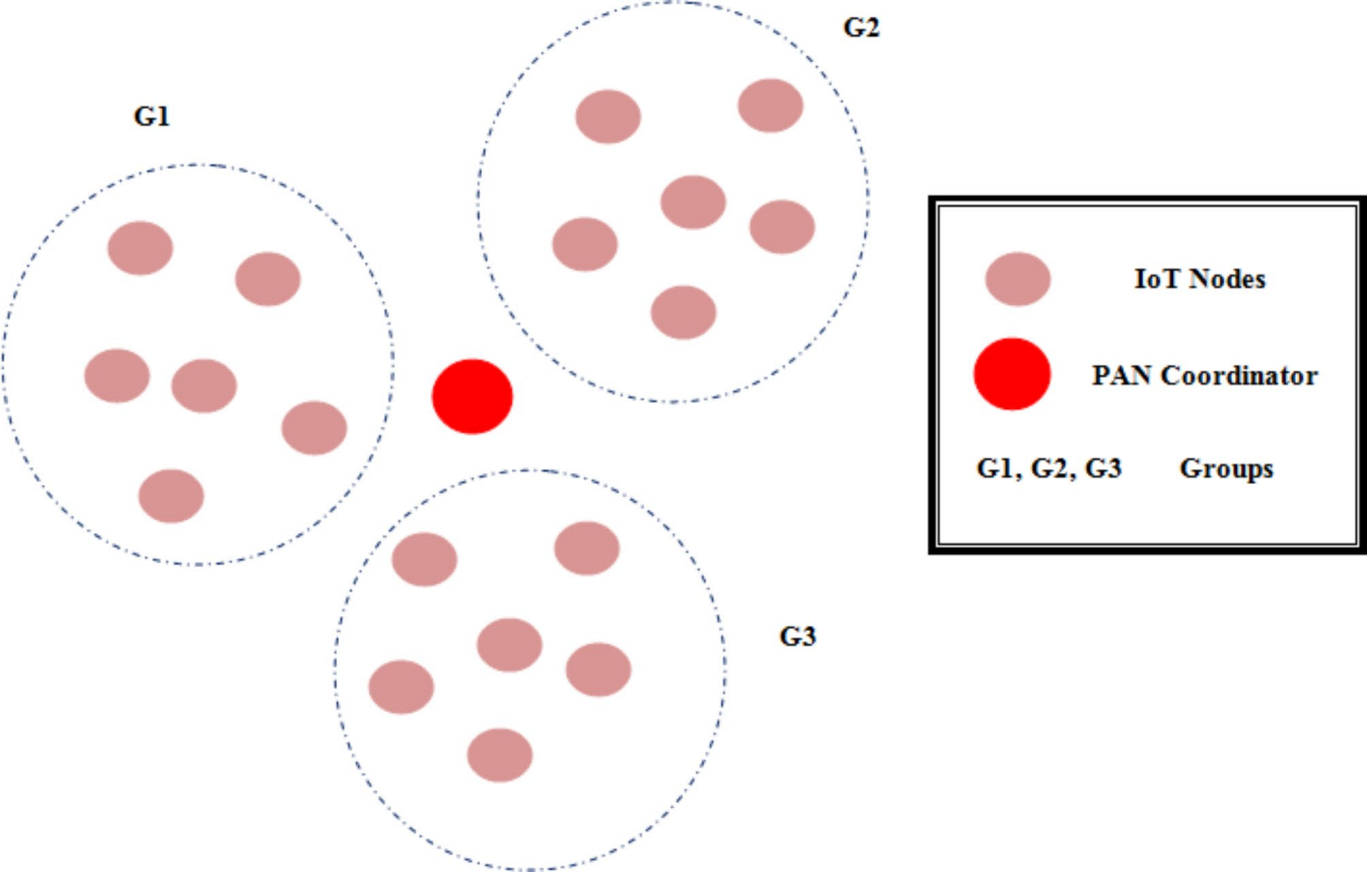
System model of IoT network.

The system model of proposed IoT network architecture is shown in Fig. 1. The IoT nodes are grouped into various clusters based on the location. The PAN Coordinator is in the middle of the network connecting all the groups. There is one control channel for node contention and data transmission.

### Group formation

In this section, the location, energy levels of nodes and traffic type are estimated to form various groups.

#### Location estimation

In this phase, the location id of a device is determined based on the distance from the PAN coordinator. Let d_pan(i)_ be the distance of a device i from the PAN coordinator. Then depending on two range thresholds D_1_ and D_2_ (D_1_ > D_2_), the locations can be defined as follows:

**Table:** 

Range	Location id
d_pan(i)_) < D_1_	L_1_
D_1_ > = d_pan(i)_ < = D_2_	L_2_
d_pan(i)_ > D_2_	L_3_

#### Energy level

Sending (*E*_*tx*_) and receiving (*E*_*rx*_) energy consumption are included in computing the total energy consumption (*E*_*tot*_) of data uploading at each sensor:

1 $${\text{E}}_{{{\text{tot}}}} = {\text{ E}}_{{{\text{tx}}}} + {\text{ E}}_{{{\text{rx}} }}$$

where

$$\mathop E\nolimits_{tx} = \sum\nolimits_{i = 1}^{h} {\mathop P\nolimits_{txi} } *L*\mathop T\nolimits_{ni} *(1 + RTrate_{i} )$$

2 $$\mathop E\nolimits_{rx} = \sum\nolimits_{i = 1}^{h - 1} {\mathop P\nolimits_{rxi} } *L*\mathop T\nolimits_{ni} *(1 + RTrate_{i} )$$

where, Ptxi and Prxi denote the sending and the receiving powers of node i, Tni is the average time required to transmit one byte of data, L is the sze of the data, RTratei denotes the data retransmission rate of node i.

Then the energy level of each node at time t is given by

3 $${\text{EL }}\left( {\text{t}} \right) \, = {\text{ E}}_{{{\text{ini}}}} {-}{\text{ E}}_{{{\text{tot}}}} \left( {\text{t}} \right)$$

where E_ini_ is the initial energy assigned to each node.

The EL of the node can be in the following modes:

- High: E_tot_ = 0,
- Moderate: E_tot_ = E_ini_ * 50%
- Low: E_tot_ = E_ini_ * 75%

#### Traffic type

The network traffic of the devices is categorized into various traffic classes based on the traffic arrival pattern, data rate and type, as shown in the following Table 1.

**Table 1:** Category of traffic classe.

Traffic class	Traffic arrival pattern	Data rate	Traffic type
C_1_	Bursty	DR1	Real time Voice
C_2_	Periodic	DR2	IoT
C_3_	Constant	DR3	Best effort

#### Group formation and priority assignment

Based on the location id, EL mode and the traffic class, the devices can be grouped into the 10 groups. Table 2 shows the details of each group along with their priority assigned.

**Table 2:** Group Priority Assignment.

Group	Location	Energy level	Traffic class	Group priority
1	L_1_	High, Moderate	C_1_	1
2	L_1_	High, Moderate	C_2_	2
3	L_1_	High, Moderate	C_3_	3
4	L_2_	High, Moderate	C_1_	4
5	L_2_	High, Moderate	C_2_	5
6	L_2_	High, Moderate	C_3_	6
7	L_3_	High, Moderate	C_1_	7
8	L_3_	High, Moderate	C_2_	8
9	L_3_	High, Moderate	C_3_	9
10	L_1_ or L_2_ or L_3_	Low	C_1_or C_2_ or C_3_	10

As illustrated in Table 2, the devices with low energy levels are assigned the least priority. The devices with high and moderate energy levels are assigned priorities according to their location and traffic type. Here the nodes from location L1 which are nearest to the PAN coordinator, are assigned highest priority of 1 to 3. In the same way, nodes from location L2 are assigned priority of 4 to 6 and nodes from location L3 are assigned priority 7 to 9.

### Estimation of contending nodes using QL-HBA

In IoT network’s control channel, the time is separated into beacon intervals. Every bbeacon interval has contention period, sensing period and data transmission period. Every contention period contains time slots for slot access request (SAR). Each IoT node initially transmits the information with the help of a chosen one time slot in the contention period. The cluster head collects the entire SAR control frames in the single-hop network. The cluster head then broadcasts a slot access confirm (SAC) control frame to all the cluster members. The eminent sensor nodes execute data transmission.

In IoT networks, based on the dynamic traffic rate, the optimum count of contention nodes within the contention period, is determined by the QL-HBA mechanism which is a hybrid of Q-learning and HBA algorithms.

#### Q-learning algorithm

Q-Learning contains 3 crucial components: Q-Table, Reward table and the Bellman Equation. The reward table is used for penalizing or rewarding an agent for its action/state compositions. The Q-Table can be assumed as experience of an agent. Initially all units of the Q-Table are assigned a zero value. Each agent gains experience as they explore the environment and update the corresponding Q-Table with the Bellman Equation.

A *Q(S*_*t*_*, A*_*t*_*)* table is maintained by the agent in *QL*. The state *S*_*t*_ of the Markov Decision Process (MDP) is observed by the agent in an IoT network for t = 1, 2, 3, ….. The agent will select an action *A*_*t*_ from the set of actions *(A).* The agent receives a reward *R(t)* and then observes the next state *S*_*t*+*1*_ after the action A_t_. The sequence of events creates the learning experience *(S*_*t,*_*A*_*t*_*,R(t), S*_*t*+*1*_*)* of the agent. The sequence of events under the *(S*_*t*_*,A*_*t*_*)* will be updated in the Q-table according to the QL function^18^.

4 $$Q(\mathop S\nolimits_{t} ,\mathop A\nolimits_{t} ) \leftarrow Q(\mathop S\nolimits_{t} ,\mathop A\nolimits_{t} ) + \alpha [\mathop R\nolimits_{t + 1} + \gamma \max Q\mathop {(\mathop S\nolimits_{t + 1} ,a) - }\limits_{{}} Q(\mathop S\nolimits_{t} ,\mathop A\nolimits_{t} )]$$

**Fig. 2:**
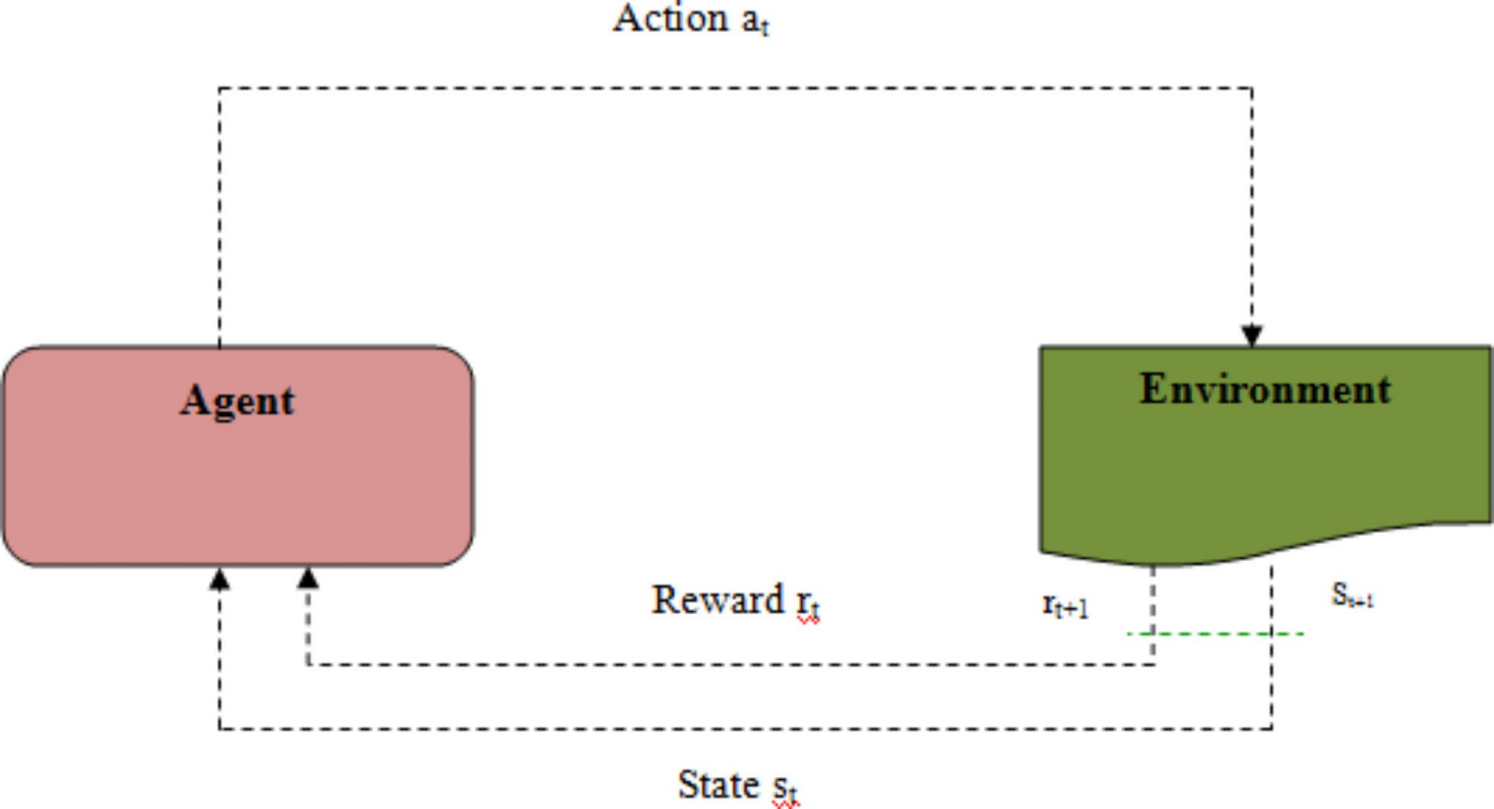
Reinforcement learning agent and environment architecture.

On the basis of state* S*_*t*_*,* the agent chooses an action. The upper limit Q-value is estimated for the next state *S*_*t*+*1*_, which can be performed based on the action *A*_*t*_. The present Q-value is updated by the upper limit Q-value. The discount factor γ’s favorable value range will be 0 < γ < 1. The learning rate α that ranged between 0 and 1 is called as the learning speed. Figure 2 shows the architecture of Q-learning.

Among the determined states, the best state will be chosen, after estimateing the degree of benefit resulting from having each achievable states for the next step. Once each step is completed, either a penalty or a reward is allocated to the agent that depends on its activity. Accordingly, Q-table is updated by the agent itself and the table for the next iteration is created with the help of the Bellman Equation.

5 $$\mathop Q\nolimits_{(t + 1)} (\mathop s\nolimits_{t} ,\mathop a\nolimits_{t} ) \leftarrow \mathop Q\nolimits_{t} (\mathop s\nolimits_{t} ,\mathop a\nolimits_{t} ) + \lambda [\mathop r\nolimits_{t + 1} + \gamma \mathop {Max\mathop Q\nolimits_{t} (\mathop s\nolimits_{t + 1} ,a) - }\limits_{{}} \mathop Q\nolimits_{t} (\mathop s\nolimits_{t} ,\mathop a\nolimits_{t} )]$$

where *s*_*t*_ and *s*_*t*+*1*_ indicate the present and the subsequent states comparatively, *a*_*t*_ indicates the present action, λ indicates learning rate value, and γ indicates the discount factor. Moreover, λ and γ range from 0 to 1. The λ defines the speed of the mechanism in learning while γ defines the knowledge the mechanism acquired from its mistakes. *r*_*t*+*1*_ indicates the next reward or penalty given for the present action. *Q*_*t*+*1*_ is the Q-value previously determined for the next state *s*_*t*+*1*_.

***Algorithm 1: The Q-Learning algorithm pseudocode.***

1. *Initialize*
2. *Set the state s and action a;*
3. *For each state s*_*i*_ *and action a*_*i*_
4. *Set Q(s*_*i*_*,a*_*i*_*)* = *0*
5. *End For*
6. *Randomly choose and initial state s*_*t*_
7. *While the terminal condition is not reached Do*
8. *Choose the best action a*_*i*_ *from the current state s*_*t*_ *from Q-values matrix*
9. *Execute action a*_*i*_ *to get the immediate reward*
10. *Find out the new state s*_*t*+*1*_
11. *Acquire the corresponding maximum Q-value for s*_*t*+*1*_
12. *Update the Q-values Table Matrix by Bellman Equation*
13. *Update the state s*_*t*_ = *s*_*t*+*1*_
14. *End While*

#### Honey Badger Algorithm (HBA)

In all Metaheuristic or optimization techniques, the search agents find the best solution based on the goal of the optimization problem. Typically, the best solution is the maximum or minimum score defined as a global optimum. In order to achieve the global optimum point, search agents have to move through all the points while ensuring no point exists with a better optimum score than the selected point. Exploration and exploitation phases are employed in metaheuristic algorithms. The search agents explore the search space during the exploration phase, whereas, they decide upon the best solution during the exploitation phase.

The mathematical expression of the proposed HBA algorithm^20^ is described in this section. Empirically, since HBA has both exploration and exploitation phases, it is defined as a global optimization algorithm. The proposed algorithm’s contains population initialization, evaluation, and parameter updates. The mathematical procedure is elaborately discussed below.

In HBA, the population of candidate solutions is indicated as:

6 $${\text{Population of candidate solutions }} = \left[ {\begin{array}{*{20}c} {\mathop x\nolimits_{11} } & {\mathop x\nolimits_{11} } & {\mathop x\nolimits_{13} } & {....} & {\mathop x\nolimits_{1D} } \\ {\mathop x\nolimits_{21} } & {\mathop x\nolimits_{22} } & {\mathop x\nolimits_{23} } & {....} & {\mathop x\nolimits_{2D} } \\ {...} & {....} & {....} & {....} & {...} \\ {\mathop x\nolimits_{n1} } & {\mathop x\nolimits_{n2} } & {\mathop x\nolimits_{n3} } & {....} & {\mathop x\nolimits_{nD} } \\ \end{array} } \right]$$

$$i^{th} {\text{position of honey badger }} = \left[ {\mathop x\nolimits_{i}^{1} ,\mathop x\nolimits_{i}^{2} ,.....,\mathop x\nolimits_{i}^{D} } \right]$$

*Step 1* (Initialization phase): The total count of honey badgers (population size N) and their corresponding positions are initialized on the basis of Eq. (1):

$x_{i} = lb_{i} + r_{1.} (ub_{i} - lb_{i} )$, r1 is a random number in (0,1).

where *x*_*i*_ represents *i*^*th*^ honey badger position that indicates a candidate solution in *N* population. *lb*_*i*_ and *ub*_*i*_ represent the lower and upper bounds of the search domain respectively.

*Step 2* (Defining intensity): Intensity (I) is related to prey’s concentration strength and distance between the prey and *i*^*th*^ honey badger. The prey’s smell intensity is indicated as Ii. The motion will become quick and vice versa when if the smell becomes high. This is represented by Inverse Square Law, which is given in the following Eqn.

$I_{i} = r_{2} .\left( {\frac{S}{{4\Pi d_{i}^{2} }}} \right)$, r2 is a random number in (0,1)

$$S = (x_{i} - x_{i + 1} )^{2}$$

7 $$d_{i} = x_{prey} - x_{i}$$

where S is source strength or concentration strength (location of prey as shown in Fig. 2). In Eq. (2), *di* denotes distance between prey and the *i*^*th*^ badger.

*Step 3* (Update density factor): The density factor (α) manages time-varying randomization for assuring smooth transformation from exploration to exploitation. The decreasing factor α is updated that reduces with repetitions for reducing randomization with time, with the help of Eq. (8):

8 $$\alpha = C.e^{{( - t/t_{\max } )}}$$

where t_max_ = maximum number of iterations, C is a constant ≥ 1 (default = 2).

*Step 4* (Escaping from local optimum): Here, the local optima regions are escaped. This algorithm utilizes a flag F which changes the search direction for providing more opportunities for agents for strictly scanning the search-space.

*Step 5* (Updating the agents’ positions): The update process of HBA position (x_new)_ is split into two phases, which are discussed as follows.

**Fig. 3:**
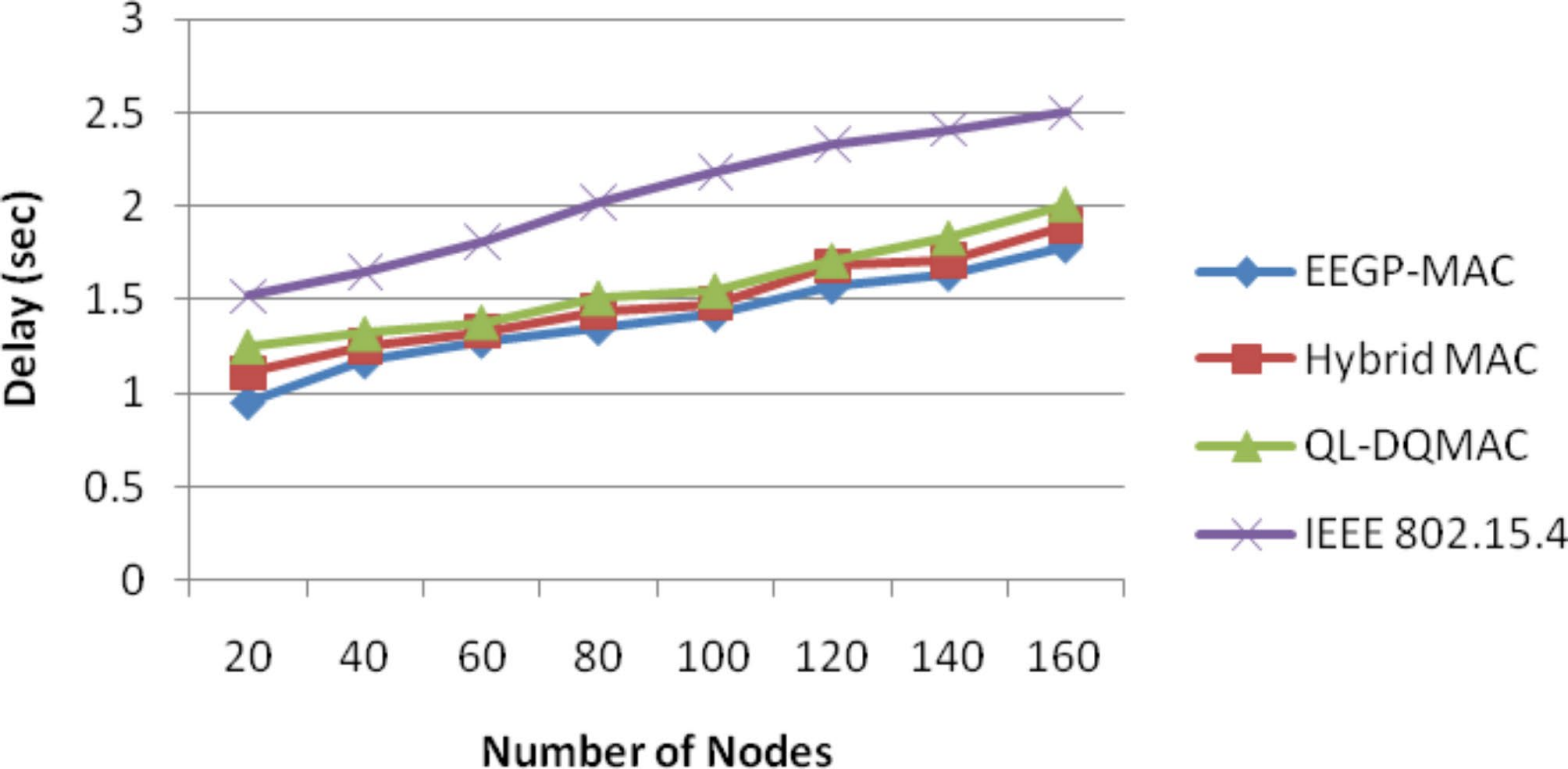
Average packet delay for nodes.

*Step 5.1*: Digging phase. During this phase, a honey badger does the action same as that of Cardioid shape as illustrated in Fig. 3. The Cardioid motion is modelled with the help of Eq. (9):

9 $$\mathop x\nolimits_{new} = \mathop x\nolimits_{prey} + Fx\beta xIx\mathop x\nolimits_{prey} + Fxr3x\alpha x\mathop d\nolimits_{i} x|\cos (2\Pi r4)x[1 - \cos (2\Pi r5)]|$$

where *x*_*prey*_ indicates the global best location that is the prey’s best location determined yet. *β* ≥ *1* (default = 6) indicates the food obtaining capacity of the honey badger. d_i_ indicates the distance between the *i*^th^ honey badger and prey), r3, r4, and r5 indicate three various random numbers ranged from 0 to 1. F operates as the flag for changing the search direction, which is estimated with the aid of Eq. (10):

10 $$F = \left\{ {\begin{array}{*{20}c} 1 & {if,r6 \le 0.5} \\ { - 1} & {otherwise} \\ \end{array} } \right.$$

r6 is a random number between 0 and 1.

During the digging phase, a honey badger is based on on smell intensity I of prey *x*_*prey*_, distance between the prey *d*_*i*_ and the badger, and time-changing search influence factor α to a great extent. In addition, while digging, a badger obtains any disturbance F that helps it in finding better prey position.

*Step 5.2*: Honey phase. The case when a honey badger follows honey guide bird to reach beehive can be simulated as Eq. (11):

11 $$\mathop x\nolimits_{new} = \mathop x\nolimits_{prey} + Fx\mathop r\nolimits_{7} x\alpha x\mathop d\nolimits_{i}$$

r7 is a random number between 0 and 1.

Where *x*_*new*_ indicates the new location of honey badger and *x*_*prey*_ indicates the position of the prey. From Eq. (11), we can see that a honey badger does search operation nearer to prey position *x*_*prey*_ determined yet according to the information regarding the distance *d*_*i*_. The search is influenced by search behavior changing over time (α). In addition, a honey badger determines the disturbance F.

#### Hybrid QL-HBA

The hybrid algorithm determines the most suitable decision made during exploitation and exploration phases. One of the following four decisions can be made:

1. If the agent is in the exploration state, it decides to stay in explore (action)
2. If the agent is in the exploitation state, it decides to stay in exploit (action)
3. If the agent is in the exploration state, it decides to transit to exploit (action)
4. If the agent is in the exploitation state, it decides to transit to explore (action)

In the hybrid algorithms, the Q-Table is also included along with the density factor and the most appropriate exploration or exploitation decision is made according to the values in this table. When the most appropriate decision is made, it is applied to the fitness function of the relevant problem or application. This whole process continues until it either finds the best possible solution or reaches the end of the defined iterations.

If the Q-value of the exploration phase is less than the Q-value of the exploitation value, Eqs. (9) and (11) are used. Similarly, if the new fitness value is better than the current fitness value, RL agent gets a positive reward (+ 1), otherwise, as a penalty the reward is negative (-1).

#### Deriving the fitness function

The traffic load at a selected node N_i_ is given by

12 $${\text{TL}}_{{\text{i}}} = \frac{{.(ar_{i}^{{}} + fr_{i}^{{}} + cr_{i} )}}{c.\eta .}$$

where c is the capacity of the radio $\eta$ is the maximum expected utilization of capacity, ar and fr are the packet arrival and forwarding rate and cr is the collision rate. Using Eq. (12), we can determine the total traffic load at node N_i_ during the superframe period T_c_.

Then the fitness function for the QL-HBA algorithm is derived as follows:

13 $${\text{Fit}}() \, = {\text{ w}}_{{1}} .{\text{ ND}}_{{\text{i}}} + \, \left( {{1} - {\text{w}}_{{2}} } \right){\text{ TL}}_{{\text{i}}}$$

where ND_i_ is the node degree which is the number of neighbours of node i, w_1_ and w_2_ are weight values ranging from 0 to 1.

Hence, the interactions between the environment at time step t and the IoT node (or agent) are discussed below:

1. On the completion of each beacon interval, the agent watches the IoT network’s environment and gathers the present number of contention nodes,* S*_*t*_.
2. The agent gets the active rate in the subsequent contention period on its own and estimates the following action *A*_*t.*_
3. The agent applies the chosen action *A*_*t*_ in the MAC protocol. The agent collects the feedback reward *R*_*t*+*1*_ from the IoT network after completing one-time step.
4. The agent moves from the existing state *S*_*t*_ to the new state *S*_*t*+*1*_.

## Results and discussion

The proposed EEGP-MAC protocol is implemented in NS3 and compared with Hybrid MAC^11^, QL-DQMAC^13^ and traditional IEEE 802.15.4 MAC protocols. The simulation settings are shown in Table 3.

**Table 3:** Simulation parameters.

Number of nodes	20 to 100
Size of the topology	50 m X 50 m
MAC protocol	802.15.4
Traffic source	CBR and exponential
Traffic flows	6
Traffic rate	50 Kb
Initial energy	15 Joules
Transmit power	0.3 watts
Receiving power	0.3 watts

## Results

### Varying the nodes

In order to check the scalability of the proposed MAC protocol, the number of nodes has been varied from 20 to 160.

Table 4 and Fig. 3 show the results of packet delay for varying the nodes. The figure depicts that the delay of EEGP-MAC is 6% lesser delay when compared to Hybrid-MAC and 11% lesser than QL-DGMAC and 32% lesser than IEE 802.15.4.

**Table 4:** Results of packet delay for nodes.

Nodes	EEGP-MAC (sec)	Hybrid MAC (sec)	QL-DQMAC (sec)	IEEE 802.15.4 (sec)
20	0.95	1.11	1.25	1.52
40	1.17	1.25	1.32	1.65
60	1.28	1.33	1.38	1.81
80	1.35	1.44	1.51	2.02
100	1.42	1.48	1.55	2.18
120	1.57	1.68	1.71	2.33
140	1.64	1.71	1.83	2.41
160	1.79	1.9	2.01	2.5

**Fig. 4:**
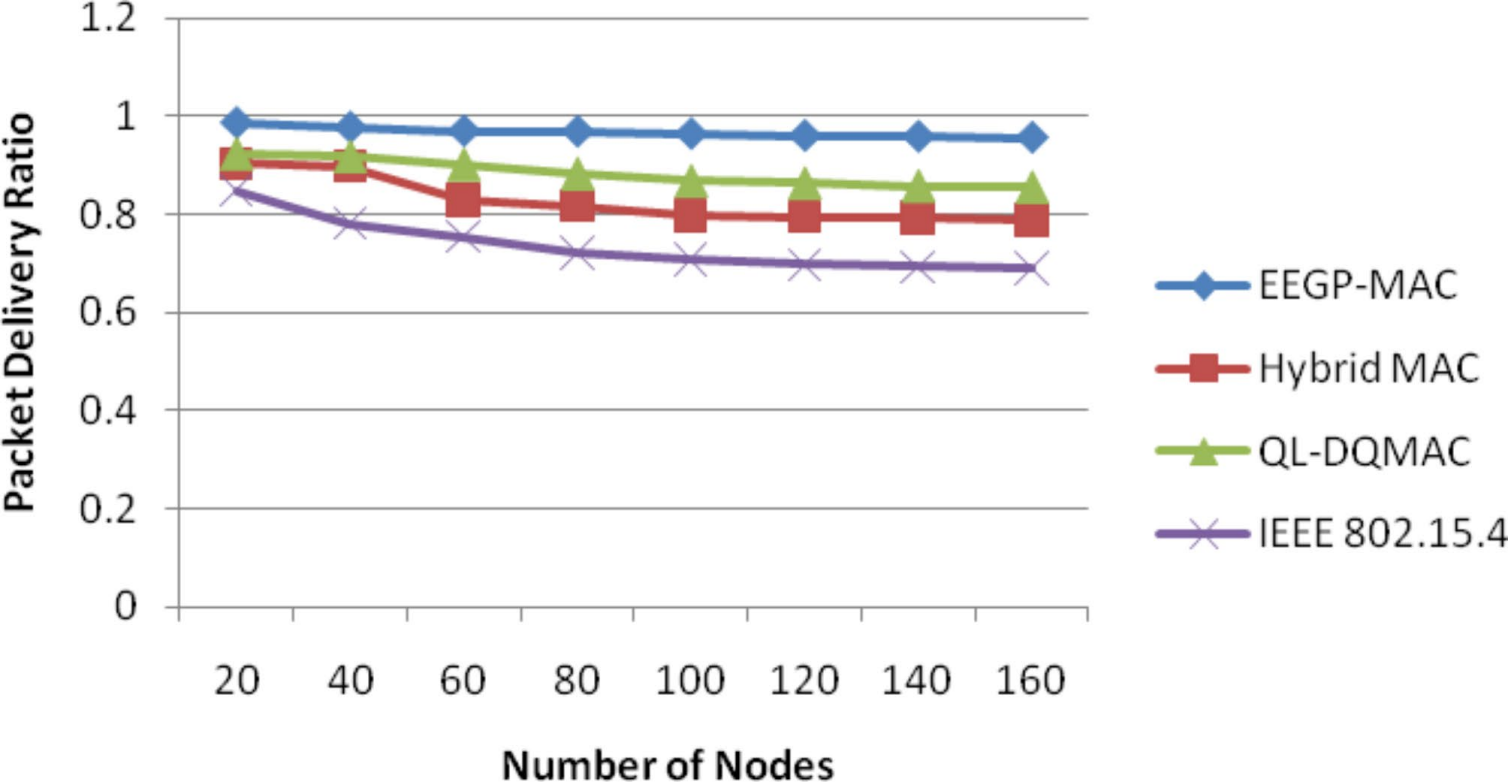
Packet delivery ratio for nodes.

Table 5 and Fig. 4 show the results of packet delivery ratio for varying the nodes. The figure depicts that the delivery ratio of EEGP-MAC is 14% higher delivery ratio when compared to Hybrid-MAC and 14% higher than QL-DGMAC and 23% higher than IEE 802.15.4.

**Table 5:** Results of packet delivery ratio for nodes.

Nodes	EEGP-MAC	Hybrid MAC	QL-DQMAC	IEEE 802.15.4
20	0.9871	0.903	0.9231	0.849
40	0.9774	0.8984	0.9174	0.7818
60	0.9701	0.8291	0.9012	0.7549
80	0.9689	0.8177	0.8829	0.7219
100	0.9643	0.7985	0.8683	0.7085
120	0.9601	0.7947	0.8637	0.6977
140	0.9583	0.7921	0.8582	0.6934
160	0.9564	0.7892	0.8553	0.6911

**Fig. 5:**
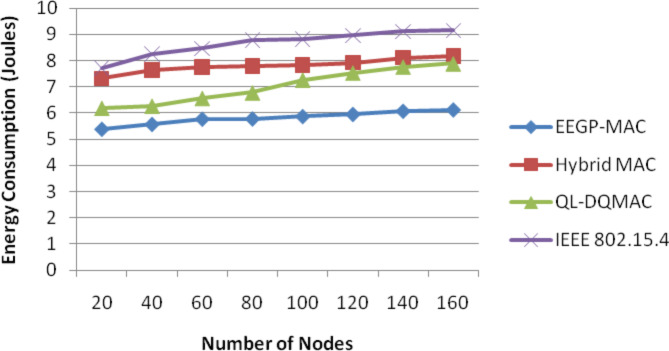
Energy consumption for nodes.

Table 6 and Fig. 5 show the results of energy consumption for varying the nodes. The figure depicts that the energy consumption of EEGP-MAC is 25% lesser energy consumption when compared to Hybrid-MAC and 16% lesser than QL-DGMAC and 32% lesser than IEE 802.15.4.

**Table 6:** Results of energy consumption for nodes.

Nodes	EEGP-MAC (Joules)	Hybrid MAC (Joules)	QL-DQMAC (Joules)	IEEE 802.15.4 (Joules)
20	5.39	7.32	6.19	7.72
40	5.57	7.64	6.27	8.24
60	5.77	7.76	6.57	8.46
80	5.78	7.79	6.78	8.79
100	5.87	7.82	7.27	8.82
120	5.95	7.92	7.64	8.96
140	6.07	8.11	7.97	9.11
160	6.12	8.18	8.09	9.15

**Fig. 6:**
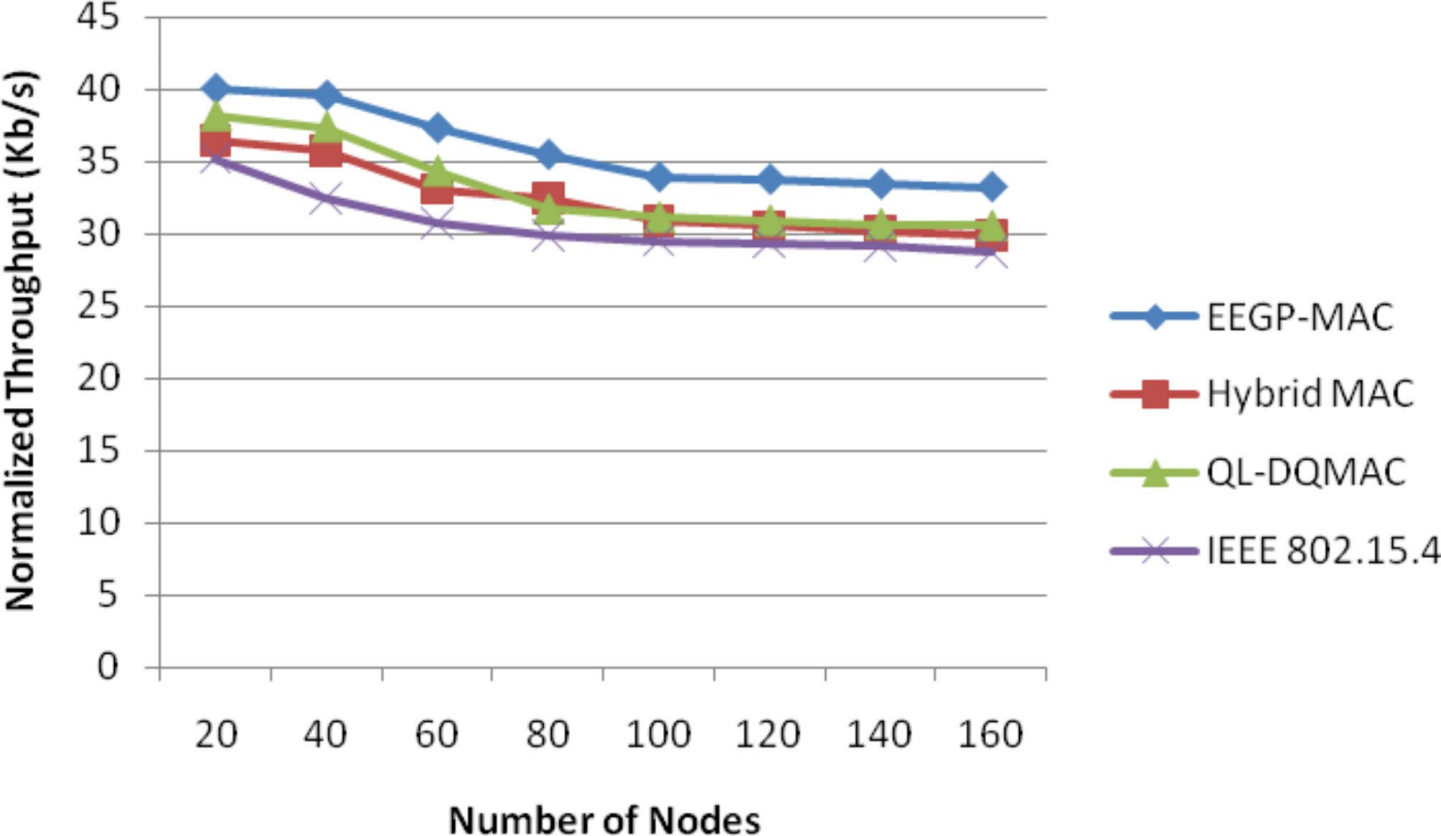
Normalized throughput for nodes.

Table 7 and Fig. 6 show the results of normalized throughput for varying the nodes. The figure depicts that the throughput of EEGP-MAC is 10% higher throughput when compared to Hybrid-MAC and 10% higher than QL-DGMAC and 15% higher than IEE 802.15.4.

**Table 7:** Results of throughput for nodes.

Nodes	EEGP-MAC (Mb)	Hybrid MAC (Mb)	QL-DQMAC (Mb)	IEEE 802.15.4 (Mb)
20	40.08	36.45	38.15	35.23
40	39.61	35.76	37.37	32.45
60	37.33	33.08	34.32	30.75
80	35.48	32.44	31.69	29.91
100	33.91	30.85	31.13	29.50
120	33.75	30.54	30.89	29.37
140	33.42	30.21	30.65	29.14
160	33.23	29.88	30.52	28.76

### Varying the traffic load

In this section, the effect of traffic load on contending nodes is analyzed by varying the Periodic (CBR) and Bursty (Exponential) traffic loads from 100 to 250 Kb/s.

## (i) For CBR Traffic scenario

In this subsection, the results of varying the CBR traffic load from 100 to 250 Kb/s are presented.

**Fig. 7:**
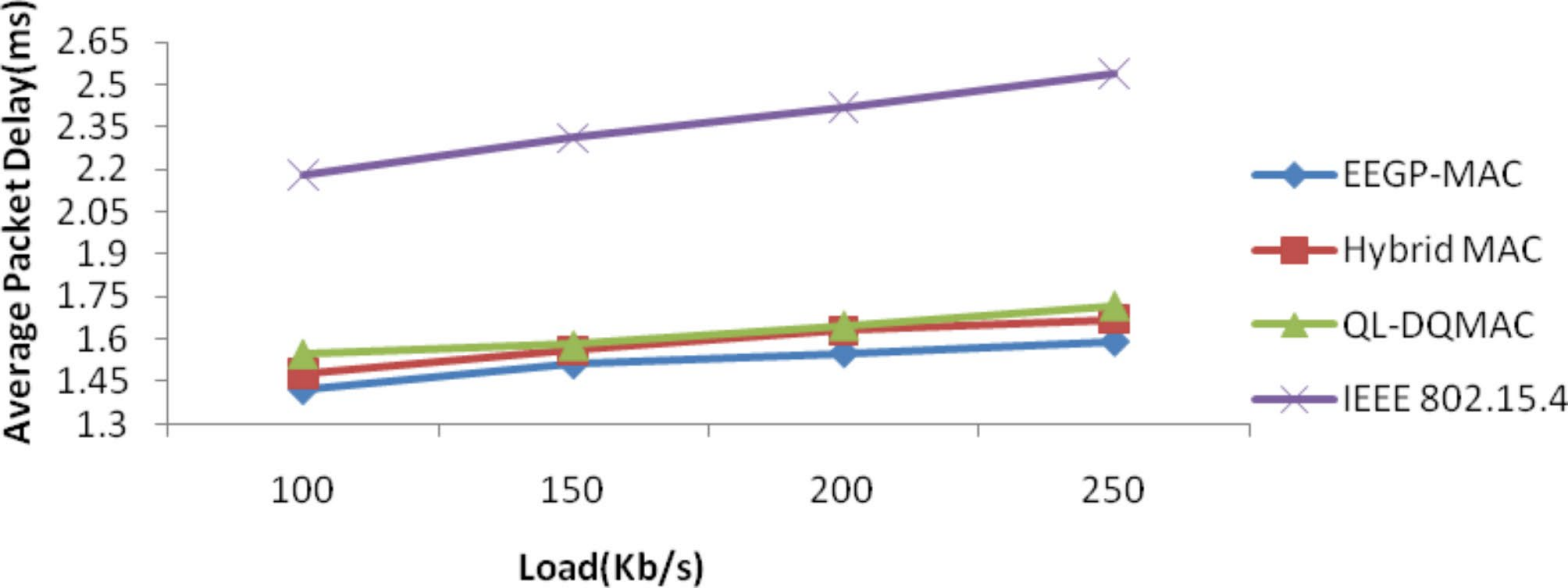
Average packet delay for CBR traffic load.

Table 8 and Fig. 7 show the results of packet delay for varying the load. The figure depicts that the delay of EEGP-MAC is 4% lesser delay when compared to Hybrid-MAC and 7% lesser than QL-DGMAC and 36% lesser than IEE 802.15.4.

**Table 8:** Results of packet delay for CBR traffic load.

Load (Kb/s)	EEGP-MAC (sec)	Hybrid MAC (sec)	QL-DQMAC (sec)	IEEE 802.15.4 (sec)
100	1.42	1.48	1.55	2.18
150	1.51	1.56	1.58	2.31
200	1.55	1.63	1.65	2.42
250	1.59	1.67	1.72	2.54

**Fig. 8:**
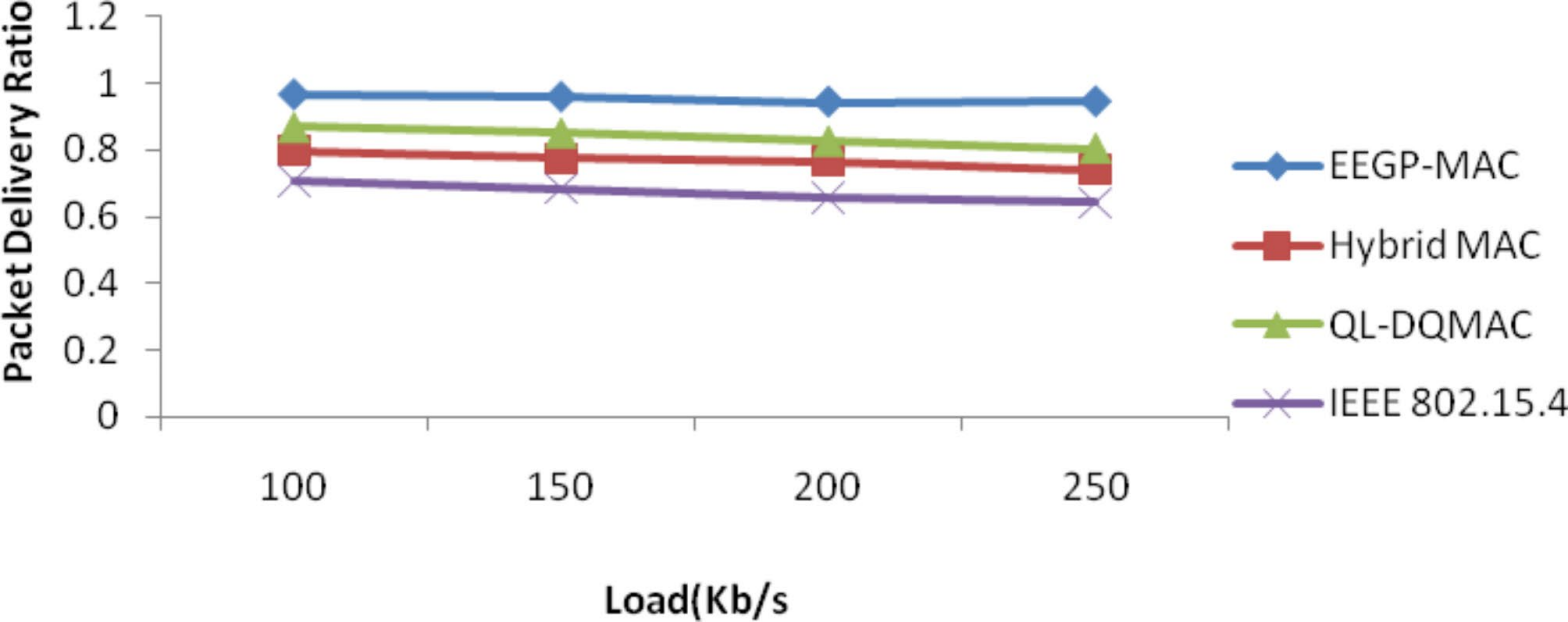
Packet delivery ratio for CBR traffic load.

Table 9 and Fig. 8 show the results of packet delivery ratio for varying the load. The figure depicts that the delivery ratio of EEGP-MAC is 19% higher delivery ratio when compared to Hybrid-MAC and 12% higher than QL-DGMAC and 29% higher than IEE 802.15.4.

**Table 9:** Results of packet delivery ratio for CBR traffic load.

Load (Kb/s)	EEGP-MAC	Hybrid MAC	QL-DQMAC	IEEE 802.15.4
100	0.9643	0.7985	0.8683	0.7085
150	0.9575	0.7734	0.8511	0.6851
200	0.9428	0.7668	0.8265	0.6583
250	0.9436	0.7414	0.8037	0.6414

**Fig. 9:**
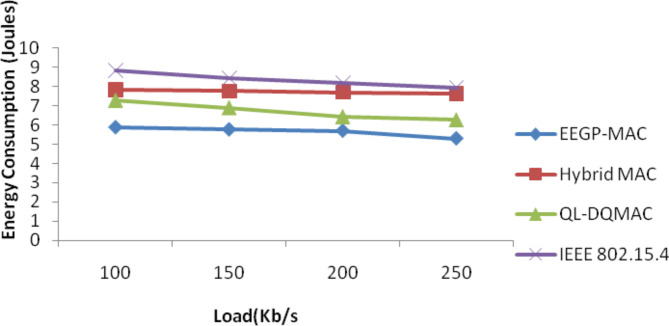
Energy consumption for CBR traffic load.

Table 10 and Fig. 9 show the results of energy consumption for varying the load. The figure depicts that the energy consumption of EEGP-MAC is 27% lesser energy consumption when compared to Hybrid-MAC and 16% lesser than QL-DGMAC and 32% lesser than IEE 802.15.4.

**Table 10:** Results of energy consumption for CBR traffic load.

Load (Kb/s)	EEGP-MAC (Joules)	Hybrid MAC (Joules)	QL-DQMAC (Joules)	IEEE 802.15.4 (Joules)
100	5.87	7.82	7.27	8.82
150	5.75	7.75	6.89	8.42
200	5.68	7.69	6.43	8.19
250	5.29	7.62	6.27	7.95

**Fig. 10:**
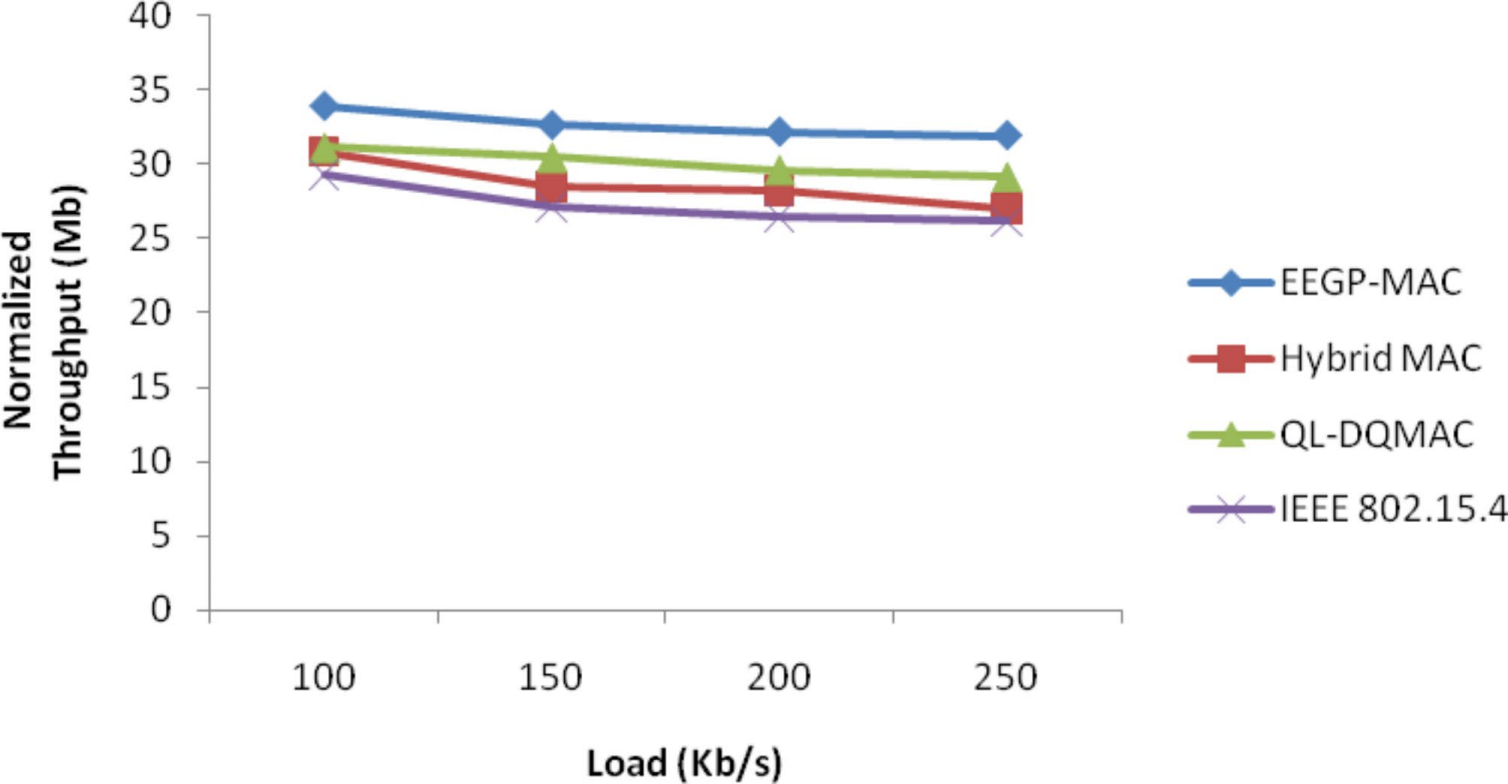
Normalized throughput for CBR traffic load.

Table 11 and Fig. 10 show the results of normalized throughput for varying the load. The figure depicts that the throughput of EEGP-MAC is 12% higher throughput when compared to Hybrid-MAC and 8% higher than QL-DGMAC and 17% higher than IEE 802.15.4.

**Table 11:** Results of throughput for CBR traffic load.

Load(Kb/s)	EEGP-MAC (Mb)	Hybrid MAC (Mb)	QL-DQMAC (Mb)	IEEE 802.15.4 (Mb)
100	33.91	30.85	31.13	29.3
150	32.62	28.51	30.47	27.11
200	32.17	28.23	29.62	26.47
250	31.93	27.04	29.14	26.15

## (ii) For Exponential Traffic scenario

In this subsection, the results of varying the exponential traffic load from 100 to 250 Kb/s are presented.

**Fig. 11:**
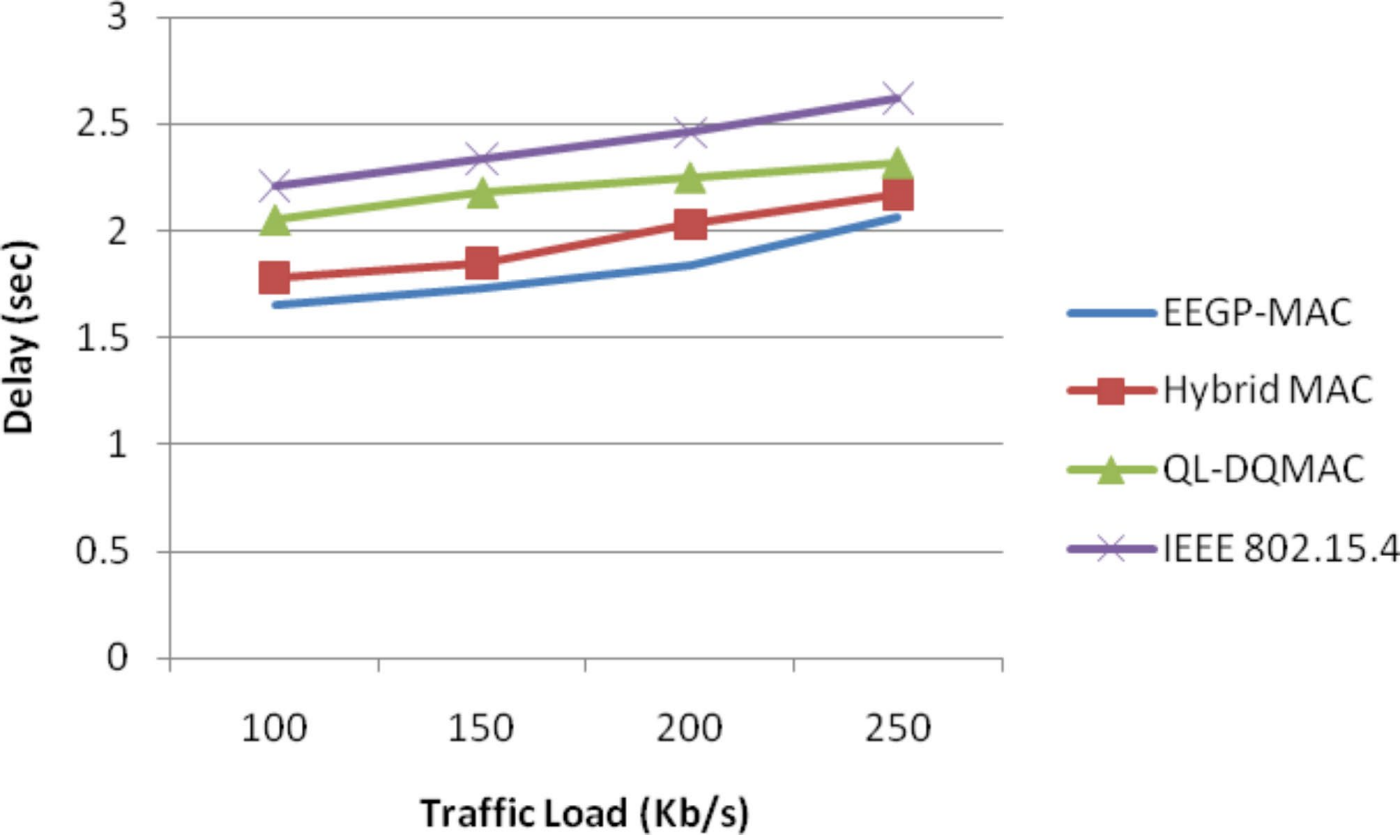
Average delay for EXP traffic load.

Table 12 and Fig. 11 show the results of packet delay for varying the load. The figure depicts that the delay of EEGP-MAC is 7% lesser delay when compared to Hybrid-MAC and 17% lesser than QL-DGMAC and 32% lesser than IEE 802.15.4.

**Table 12:** Results of packet delay for EXP traffic load.

Load (Kb/s)	EEGP-MAC (sec)	Hybrid MAC (sec)	QL-DQMAC (sec)	IEEE 802.15.4 (sec)
100	1.65	1.78	2.05	2.21
150	1.73	1.85	2.18	2.34
200	1.84	2.03	2.25	2.46
250	2.06	2.17	2.32	2.62

**Fig. 12:**
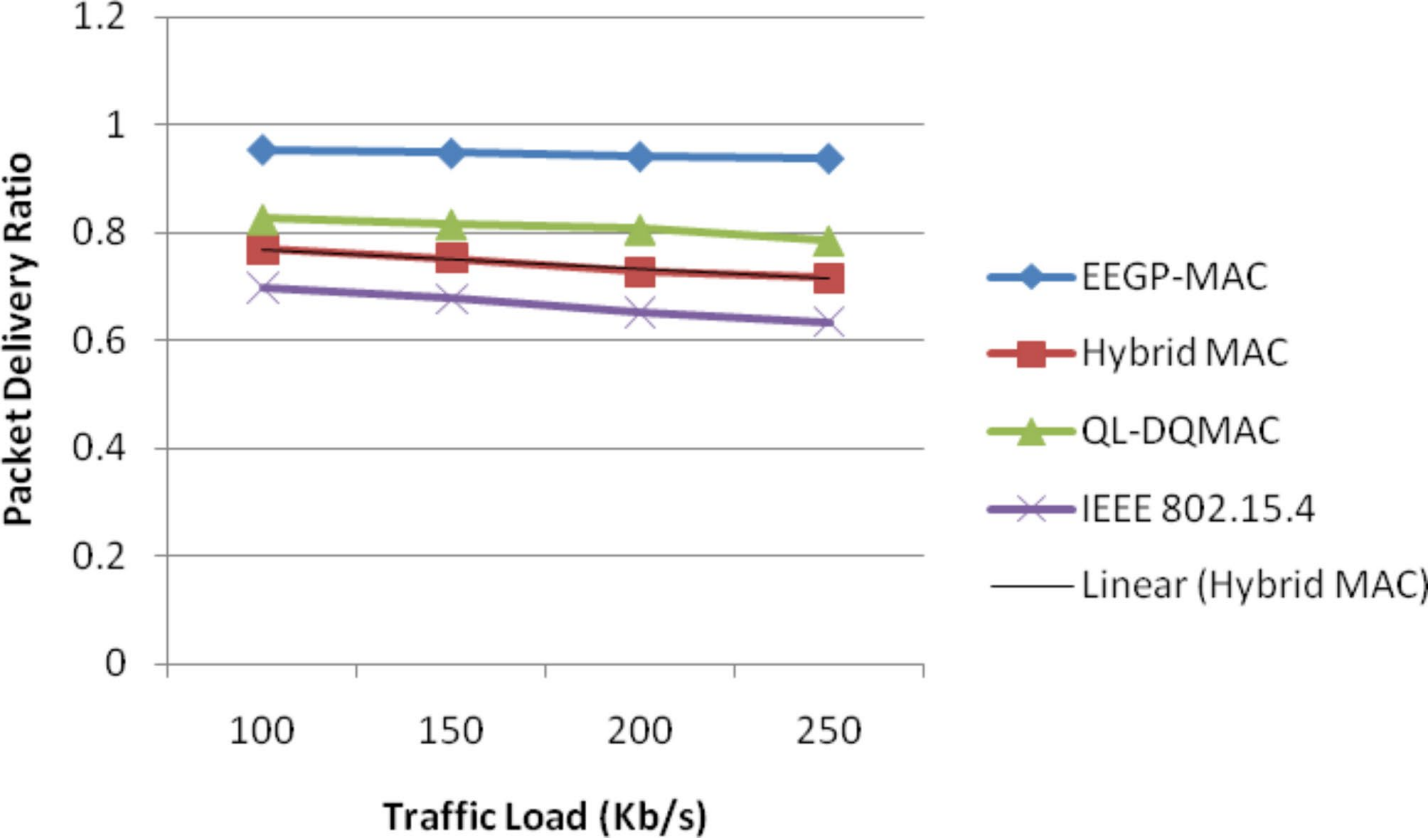
Packet delivery ratio for EXP traffic load.

Table 13 and Fig. 12 show the results of packet delivery ratio for varying the load. The figure depicts that the delivery ratio of EEGP-MAC is 21% higher delivery ratio when compared to Hybrid-MAC and 21% higher than QL-DGMAC and 29% higher than IEE 802.15.4.

**Table 13:** Results of packet delivery ratio for EXP traffic load.

Load (Kb/s)	EEGP-MAC	Hybrid MAC	QL-DQMAC	IEEE 802.15.4
100	0.9542	0.7685	0.8253	0.6975
150	0.9477	0.7524	0.8161	0.6771
200	0.9418	0.7267	0.8065	0.6523
250	0.9382	0.7154	0.7857	0.6341

**Fig. 13:**
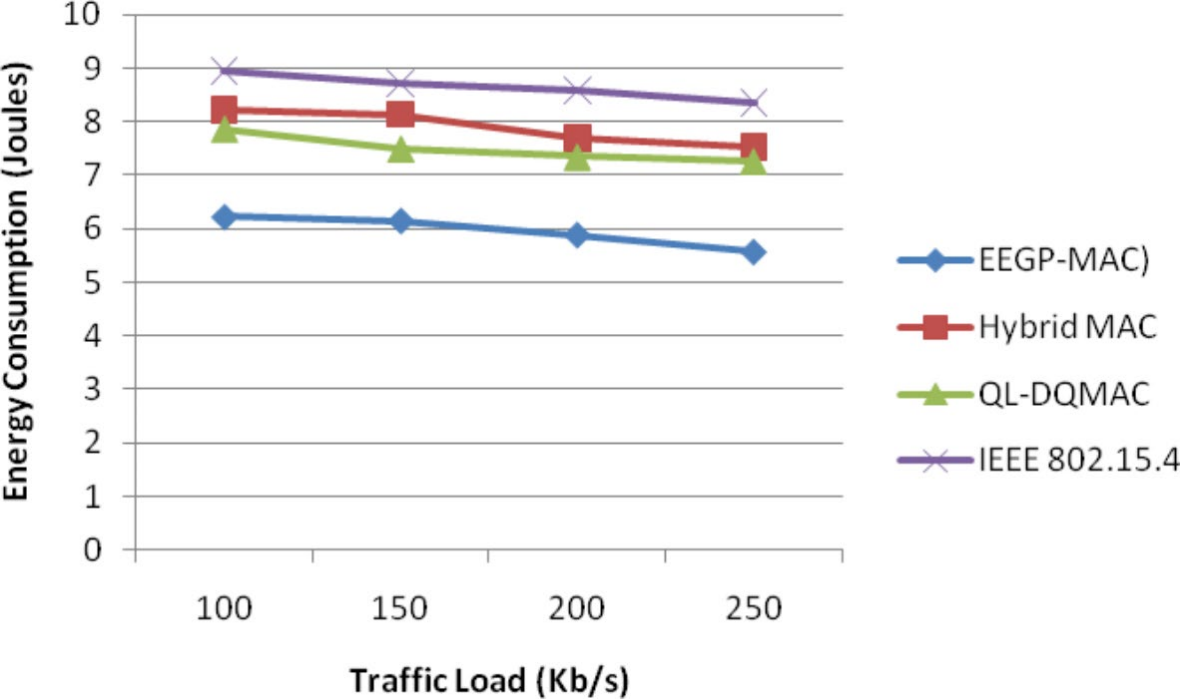
Energy consumption for EXP traffic load.

Table 14 and Fig. 13 show the results of energy consumption for varying the load. The figure depicts that the energy consumption of EEGP-MAC is 32% lesser energy consumption when compared to Hybrid-MAC and 20% lesser than QL-DGMAC and 45% lesser than IEE 802.15.4.

**Table 14:** Results of Energy consumption for EXP traffic load.

Load (Kb/s)	EEGP-MAC (Joules)	Hybrid MAC (Joules)	QL-DQMAC (Joules)	IEEE 802.15.4 (Joules)
100	6.22	8.22	7.87	8.95
150	6.15	8.13	7.5	8.72
200	5.88	7.69	7.35	8.59
250	5.56	7.52	7.27	8.35

**Fig. 14:**
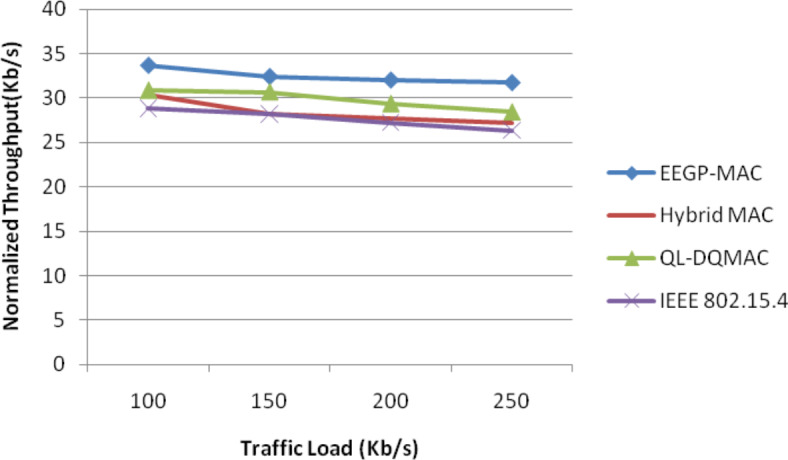
Normalized throughput for EXP traffic load.

Table 15 and Fig. 14 show the results of normalized throughput for varying the load. The figure depicts that the throughput of EEGP-MAC is 12% higher throughput when compared to Hybrid-MAC and 12% higher than QL-DGMAC and 14% higher than IEE 802.15.4.

**Table 15:** Results of Throughput for EXP traffic load.

Load(Kb/s)	EEGP-MAC (Mb)	Hybrid MAC (Mb)	QL-DQMAC (Mb)	IEEE 802.15.4 (Mb)
100	33.7	30.35	30.84	28.86
150	32.45	28.21	30.67	28.19
200	32.05	27.75	29.35	27.23
250	31.76	27.24	28.41	26.35

## Discussion

The Hybrid-MAC combines the TDMA and CSMA MAC protocols so that only a specific set of nodes will contend for the TDMA slots. However, it didn’t consider the traffic arrival pattern and load while selecting the contenders. Hence the associated delay will be higher. Moreover, it didn’t consider the collisions from neighbour nodes, resulting in reduced throughput and increased energy consumption.

The QL-DGMAC selects the contending nodes based on traditional Q-learning only, hence the corresponding accuracy of decisions may not be high. Moreover, it didn’t consider the traffic arrival pattern and load while selecting the contenders.

When compared to QL-DGMAC and Hybrid-MAC, the proposed EEGP-MAC assigns the group priority based on traffic patterns and energy. It also considers the traffic load while selecting the contenders. Since the Q-learning algorithm is optimized by HBA, the decisions will be accurate resulting in appropriate number of contenders, without any delay. Hence as seen from the results depicted in the previous section, the EEGP-MAC attains lowest delay, minimized energy consumption and highest throughput, in case of both periodic and bursty traffic scenarios.

## Conclusion

In this paper, EEGP-MAC protocol has been proposed for IoT Networks. It makes use of Q-learning method in HBA. In group priority assignment phase, a combined metric will be derived in terms of location, energy levels and the traffic type parameters. Then a priority will be assigned to each group based on the combined metric. From each group, the optimal number of contention nodes is selected using hybrid QL-HBA algorithm. The fitness function is derived in terms of the number of neighbours and total traffic loads of the nodes. Then transmission slots will be allotted to the group according to their group priority. The proposed EEGP-MAC protocol is implemented in NS3 and compared with Hybrid MAC, QL-DGMAC and traditional IEEE 802.15.4 MAC protocols. Simulation results have shown that EEGP-MAC attains least delay and energy consumption with maximized throughput and packet delivery ratio.

## Data Availability

The authors confirm that the data supporting the findings of this study are available within the article.
